# Comparison of Long-Term Oncological Outcomes of Intravesical Bacillus Calmette–Guérin Versus Gemcitabine in Treatment-Naïve Non-Muscle-Invasive Bladder Cancer with Intermediate and High Risk: A Multicenter Retrospective Analysis

**DOI:** 10.3390/jcm15103890

**Published:** 2026-05-18

**Authors:** Kyung Hwan Kim, Byeong Jin Kang, Chan Ho Lee, Soodong Kim, Ja Yoon Ku, Hong Koo Ha

**Affiliations:** 1 Department of Urology, Pusan National University Hospital, Pusan National University School of Medicine, Busan 49241, Republic of Korea; bravekim80@naver.com (K.H.K.); uropean86@gmail.com (B.J.K.); 2 Biomedical Research Institute, Pusan National University Hospital, Busan 49241, Republic of Korea; 3 Department of Urology, Busan Paik Hospital, Inje University College of Medicine, Busan 47392, Republic of Korea; leechanho@naver.com; 4 Department of Urology, Dong-A University College of Medicine, Busan 49201, Republic of Korea; urotan@dau.ac.kr; 5 Department of Urology, Dongnam Institute of Radiological and Medical Sciences, Busan 46033, Republic of Korea; pnumed@pusan.ac.kr

**Keywords:** Bacillus Calmette–Guérin, adjuvant therapy, non-muscle-invasive bladder cancer, adverse effects, gemcitabine, recurrence-free survival, progression-free survival

## Abstract

**Background/Objectives:** Although intravesical Bacillus Calmette–Guérin (BCG) is an established adjuvant therapy for non-muscle-invasive bladder cancer (NMIBC), chronic global shortages and adverse events (AEs) can occur. Thus, intravesical gemcitabine has been used as an alternative. We compared the long-term oncological outcomes and safety profiles of BCG and gemcitabine in treatment-naïve patients with intermediate- and high-risk NMIBC. **Methods:** Patients with intermediate- and high-risk NMIBC (*n* = 477) received adjuvant intravesical induction and maintenance therapy with intravesical BCG (*n* = 361) or gemcitabine (*n* = 116) and their data were collected retrospectively. **Results:** Compared with the gemcitabine group, the BCG group had significantly higher proportions of patients with T1 stage, high-grade tumors, high-risk tumors, and longer median follow-up duration. Over a median 36-month observation period, the BCG group exhibited significantly better recurrence-free survival (RFS) and high-grade RFS (HG-RFS) than the gemcitabine group. In the propensity score–matched high-risk population, BCG also outperformed gemcitabine in RFS and HG-RFS. BCG therapy was identified as a potent protective predictor, reducing the risk of recurrence and high-grade recurrence by 65% and 66%, respectively, in the total cohort, and by 69% and 71%, respectively, in the propensity score-matched high-risk subgroup. No significant differences were observed in the frequency of grade ≥ 3 AEs between BCG and gemcitabine. **Conclusions:** Intravesical BCG is strongly associated with superior oncological outcomes over gemcitabine in intermediate- and high-risk NMIBC. The results of this study offer pivotal practice-based insights to guide clinical strategies for managing NMIBC.

## 1. Introduction

Globally, bladder cancer is the ninth most frequently diagnosed malignancy, accounting for 614,298 new cases in 2022 [1]. Approximately 75% of patients with bladder malignancy present with non-muscle-invasive bladder cancer (NMIBC) at diagnosis [2]. Despite generally favorable survival outcomes, NMIBC imposes a substantial socioeconomic burden and negatively affects patient quality of life because of frequent recurrences and the requirement for intensive surveillance [3,4,5,6].

Current clinical guidelines mandate adjuvant intravesical instillation after the transurethral resection of bladder tumors (TURB) to manage patients with intermediate- and high-risk NMIBC. Intravesical chemotherapy or 1 year of full-dose Bacillus Calmette–Guérin (BCG) therapy is recommended for patients with intermediate-risk disease, whereas full-dose intravesical BCG for 1–3 years is recommended for those with high-risk disease [7,8]. BCG with maintenance therapy demonstrates superior efficacy to chemotherapy agents for high-risk disease; however, chemotherapy alternatives, including mitomycin C (MMC) and gemcitabine, achieve comparable recurrence control in intermediate-risk disease [9,10,11].

While intravesical BCG remains the preferred adjuvant therapy for NMIBC, chronic global shortages have resulted in considerable treatment delays and suboptimal oncologic outcomes [12,13,14]. In response to these supply constraints, intravesical gemcitabine has emerged as a widely adopted alternative in the Republic of Korea, particularly following its inclusion in national health insurance coverage in February 2022 [15]. Additionally, although NMIBC is associated with favorable overall survival rates, intensive surveillance is mandatory because of frequent recurrence and risk of progression [16]. As residual tumors are identified in nearly half of the patients following TURB, adjuvant intravesical treatment is strongly recommended [17].

In the current landscape of NMIBC management, extensive research is actively underway to identify effective alternatives to intravesical BCG or to enhance its efficacy in the adjuvant setting. Promising ongoing investigations include the SunRISe-3 trial (TAR-200 plus cetrelimab), the BRIDGE trial (gemcitabine-docetaxel), and the CORE-008 trial (cretostimogene grenadenorepvec) [18,19,20]. Additionally, the phase 3 CREST trial demonstrated that adding subcutaneous sasanlimab, an anti-programmed cell death-1 monoclonal antibody, to standard BCG therapy substantially improved event-free survival in BCG-naïve high-risk NMIBC [18]. While several comparative studies exist for intermediate- and high-risk NMIBC, large-scale real-world data with long-term follow-up remain scarce. Therefore, this study aimed to evaluate the real-world treatment patterns and compared the oncological outcomes and safety profiles of intravesical therapy with BCG and gemcitabine.

## 2. Materials and Methods

### 2.1. Study Design and Population

This multicenter retrospective analysis was conducted across three tertiary referral medical centers in Busan, Republic of Korea. We identified treatment-naïve 477 patients aged ≥18 years who underwent TURB for NMIBC between April 2017 and April 2025.

Inclusion criteria were as follows: (1) patients diagnosed with intermediate- or high-risk NMIBC after the initial or second TURB based on the American Urological Association (AUA) risk stratification; (2) pathological confirmation of urothelial carcinoma (UC) as the primary component; and (3) initiation of intravesical induction therapy using either BCG or gemcitabine within 3 months of TURB, consisting of at least four sessions of the induction phase, followed by maintenance phase therapy. Patients were excluded from the final cohort if they had a documented history of bladder or upper tract tumors or if insufficient data were available for analysis. Additionally, patients whose follow-up lasted <6 months after the initial or second TURB were excluded from the analysis.

Treatment allocation was primarily guided by AUA risk stratification, with BCG and gemcitabine preferentially administered to high-risk and intermediate-risk patients, respectively. However, treatment selection was also influenced by institutional BCG availability during the study period, as BCG shortages occasionally necessitated the use of gemcitabine as an alternative in some high-risk patients, or BCG was administered to intermediate-risk patients at the physician’s discretion.

### 2.2. Treatment Protocol

Patients received intravesical instillations of either BCG (Oncotice^®^, a live attenuated *Mycobacterium bovis* strain; Merck & Co., Inc., Rahway, NJ, USA) at a dose of 12.5 mg diluted in 50 mL of normal saline (NS), or gemcitabine (Gemzar^®^, Boryung Pharmaceutical Co., Ltd., Seoul, Republic of Korea; Gemcit^®^, Dong-A ST Co., Ltd., Seoul, Republic of Korea) at 2000 mg in an equal volume of NS. Both treatment arms initiated a 6-session induction phase between 2 and 12 weeks after TURB, but preferably within 6 weeks. Following induction, the gemcitabine group adhered to a 1-year maintenance protocol with monthly instillations. In contrast, the BCG maintenance schedule was tailored to the individual risk of recurrence or progression, spanning 1–3 years and involving 3-week cycles (once weekly) administered at 3, 6, 12, 18, 24, 30, and 36 months after TURB. A second TURB was performed 2–6 weeks after the initial procedure in patients at a high risk of recurrence or progression.

Intravesical treatment was discontinued in cases of severe adverse events (AEs), disease recurrence or progression, patient refusal of therapy, or at the physician’s discretion. Dose reduction was not performed.

### 2.3. Data Collection and Definitions

Baseline patient characteristics, including age, anthropometric measurements (height, weight, and body mass index), smoking history, and comorbidities, were extracted from medical records. Surgical and pathological data included the date of the initial TURB, tumor stage (T stage), tumor grade, multiplicity, maximal size of the tumor, carcinoma in situ (CIS) component, presence of muscularis propria in the specimen, lymphovascular or prostatic urethral invasion, and AUA risk stratification. Additionally, we recorded the dates of the second TURB, administration of a single immediate instillation, any or high-grade recurrence, and progression. The patterns and AEs of BCG or gemcitabine instillation and the date of the last follow-up were also documented.

Recurrence was defined as the pathological confirmation of UC via cystoscopic biopsy or TURB. Progression was defined as a pathological elevation in the T stage or tumor grade. Recurrence-free survival (RFS) was defined as the time from the initial or second TURB to recurrence, and progression-free survival (PFS) was defined as the time to progression. Patients were considered to have received adequate BCG therapy after receiving at least five induction and two maintenance instillations (in one or more cycles) within a 6-month window [21].

### 2.4. Surveillance Protocol

For intermediate-risk patients, follow-up cystoscopy was performed quarterly throughout the initial year post-TURB, and subsequently, it shifted to a semi-annual schedule. For high-risk patients, quarterly cystoscopy was performed for the first 2 years, followed by a semi-annual examination. All patients underwent annual computed tomography of the abdomen and pelvis.

### 2.5. Statistical Analysis

Continuous variables were assessed using the Student *t*-test or Mann–Whitney U test, and categorical variables were compared using the chi-square test or Fisher exact test. Propensity score matching (PSM) was performed within the high-risk subgroup using a logistic regression model incorporating age, T stage, tumor grade, single immediate instillation, and smoking history, with a 2:1 nearest-neighbor algorithm, yielding a matched cohort of 273 patients (BCG group = 182, gemcitabine group = 91). Kaplan–Meier analysis with the log-rank test was performed to estimate and compare RFS, high-grade recurrence-free survival (HG-RFS), and PFS between the BCG and gemcitabine groups. Univariate and multivariate Cox proportional hazards regression analyses were performed to identify independent risk factors for RFS and HG-RFS. For all analyses, a *p*-value < 0.05 was defined as the threshold for statistical significance, and statistical analyses were performed using SPSS Statistics for Windows (version 28.0; IBM Corp., Armonk, NY, USA).

### 2.6. Cohort Size Analysis

A post hoc power analysis was conducted to evaluate the adequacy of the cohort size. The study population of 477 cases (BCG arm, *n* = 361; gemcitabine arm, *n* = 116) provided a statistical power of 83.7% at a significance level of 0.05.

### 2.7. Use of Artificial Intelligence-Assisted Tools

In the preparation of this manuscript, artificial intelligence-assisted tools, including Google Gemini (version 2.5 Pro; Google LLC, Mountain View, CA, USA), Claude (claude-sonnet-4.6; Anthropic PBC, San Francisco, CA, USA), and Elicit (Elicit Inc., San Francisco, CA, USA; https://elicit.com), were used to support literature searches and assist with the refinement of English language. All contents, data interpretation, and conclusions remain the sole responsibility of the authors.

### 2.8. Ethical Statements

This study was conducted in accordance with the ethical standards approved by the Institutional Review Board of Pusan National University Hospital (No. 2601-003-158; date of approval, 12 January 2026) and the ethics committees of all participating institutions. Furthermore, the requirement for informed consent was waived by the respective boards due to the retrospective study design. This study was conducted and reported in accordance with the STROBE (Strengthening the Reporting of Observational Studies in Epidemiology) guidelines.

## 3. Results

**Figure 1:**
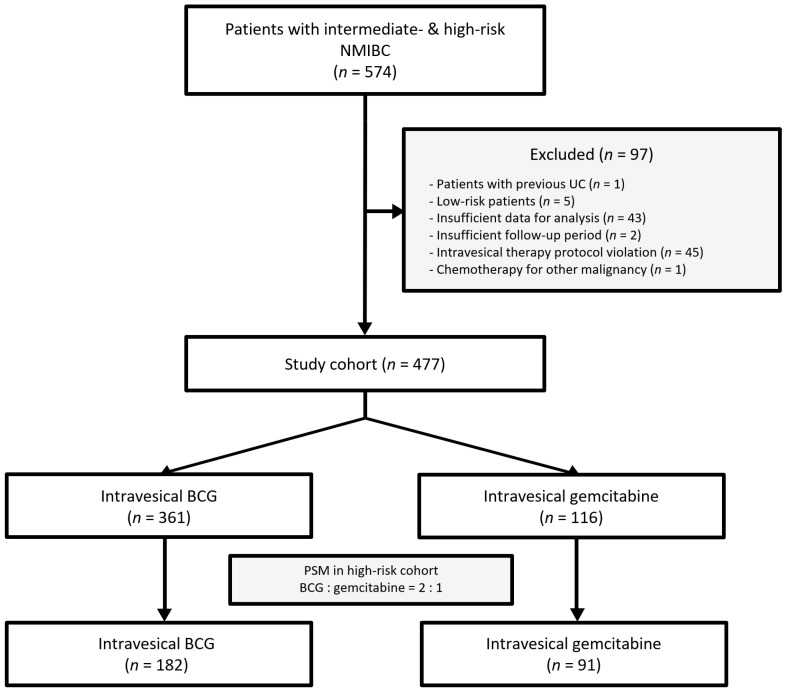
Patient selection flow chart. NMIBC, non-muscle-invasive bladder cancer; UC, urothelial carcinoma; BCG, Bacillus Calmette–Guérin; PSM, propensity score matching.

Data from 477 patients with NMIBC treated with intravesical therapy following an initial or second TURB were analyzed (Figure 1). The cohort included 361 patients who received intravesical BCG and 116 patients who received gemcitabine (Table 1). The BCG group had significantly higher proportions of patients with T1 stage tumors, high-grade tumors, high-risk tumors, and second TURB; conversely, the gemcitabine group showed higher rates of muscle inclusion in the specimen and a single immediate instillation after the initial TURB. A significantly longer median follow-up period was observed in the patients who received BCG than in those treated with gemcitabine (45.0 months versus [vs.] 24.0 months; *p* < 0.001).

Intravesical therapy-related factors are shown in Table 2. The median number of induction and maintenance instillations of BCG were both 6.0 (interquartile range [IQR]: 6.0–6.0 and 2.0–12.0, respectively), and adequate BCG therapy was achieved in 286/361 (79.2%) patients. For the gemcitabine group, median numbers of induction and maintenance instillations were 6.0 (IQR: 6.0–6.0) and 8.0 (IQR: 4.0–10.0), respectively. Intravesical BCG treatment was maintained for a longer duration than gemcitabine treatment (12.9 months vs. 10.4 months; *p* < 0.001). Compared with the gemcitabine group, the BCG group experienced significantly higher rates of irritative voiding symptoms (dysuria, frequency, and urgency: 28.5% vs. 14.7%, *p* = 0.003) and hematuria (10.2% vs. 2.6%, *p* = 0.007). Although systemic complications (fever and urosepsis) were rare in both the groups, gastrointestinal side effects (nausea and vomiting) were uniquely observed in the gemcitabine group (3.4% vs. 0.0%, *p* = 0.003). The occurrence of severe AEs (grade 3 or higher) was comparable between the BCG and gemcitabine groups, with no statistical disparity observed.

**Figure 2:**
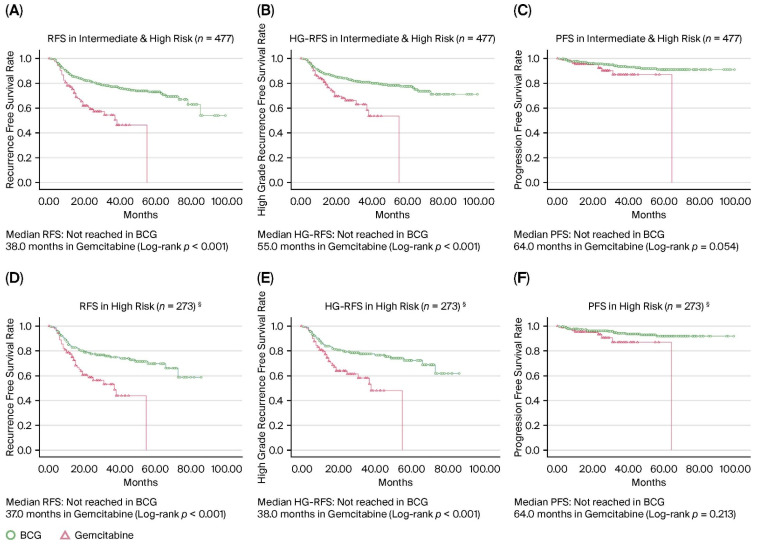
Comparison of recurrence-, high-grade recurrence-, and progression-free survival rates between intravesical BCG and gemcitabine therapies. Recurrence-free survival rate (**A**), high-grade recurrence-free survival rate (**B**), and progression-free survival rate (**C**) in intermediate- and high-risk patients (*n* = 477). Recurrence-free survival rate (**D**), high-grade recurrence-free survival rate (**E**), and progression-free survival rate (**F**) in high-risk patients (*n* = 273; propensity score-matched cohort). RFS, recurrence-free survival; HG-RFS, high-grade recurrence-free survival; PFS, progression-free survival; BCG, Bacillus Calmette–Guérin. § Propensity score–matched cohort.

Oncological outcomes for both treatment arms were analyzed using the Kaplan–Meier method, and the resulting survival data are shown in Figure 2. Among the 477 patients, BCG demonstrated significant superiority to gemcitabine in terms of RFS and high-grade RFS (HG-RFS). While the median RFS and HG-RFS were not reached in the BCG arm, the gemcitabine arm had a median RFS of 38.0 months (95% confidence interval [CI]: 30.7–45.4; log-rank *p* < 0.001) and a median HG-RFS of 55.0 months (95% CI: not estimable; log-rank *p* < 0.001). Regarding disease progression, the BCG group showed a longer PFS than the gemcitabine group, although this disparity failed to achieve statistical significance (not reached vs. 64.0 months, log-rank *p* = 0.054).

To validate the robustness of the primary findings, PSM was performed within the high-risk subgroup, yielding a matched cohort of 273 patients (BCG group = 182, gemcitabine group = 91). The baseline characteristics of the high-risk patients before and after PSM are presented in Table 3. Prior to matching, single immediate instillation showed a statistically significant imbalance between the groups (BCG, 17.2% vs. gemcitabine, 30.8%; SMD = 0.321, *p* = 0.007), and smoking history demonstrated a moderate degree of imbalance (BCG, 37.9% vs. gemcitabine, 54.9%; SMD = 0.346), although this did not reach statistical significance (*p* = 0.076). Following 2:1 nearest-neighbor PSM, all PSM covariates achieved substantially improved balance.

In the 2:1 propensity score-matched high-risk cohort, BCG continued to demonstrate superior oncological outcomes compared with gemcitabine. The median RFS was significantly longer in the BCG arm than in the gemcitabine arm (not reached vs. 37.0 months; log-rank *p* < 0.001). Similarly, the median HG-RFS was not reached in the BCG group, whereas it was 38.0 months in the gemcitabine group (log-rank *p* < 0.001). Concerning PFS, the median PFS was not reached in the BCG group, whereas it was 64.0 months in the gemcitabine group; however, no statistically significant difference was observed between the groups in the matched cohort (log-rank *p* = 0.213). No distinct survival benefit was identified for either agent among intermediate-risk patients, with similar rates of RFS, HG-RFS, and PFS observed across both treatment arms (all log-rank *p* > 0.05) (Figure S1).

In the total cohort of intermediate- and high-risk cases (*n* = 477), the multivariate Cox proportional hazards model identified age, smoking history, number of tumors, and tumor size as independent predictors of recurrence and high-grade recurrence (all *p* < 0.05; Table 4). Intravesical BCG therapy was identified as an independent protective factor suppressing recurrence and high-grade recurrence, with hazard ratios of 0.35 and 0.34, respectively (both *p* < 0.001).

In the propensity score-matched high-risk cohort (*n* = 273), the number of tumors and intravesical BCG therapy remained consistently significant independent predictors of RFS and HG-RFS (all *p* < 0.05; Table 5). The tumor size was identified as a significant independent predictor of HG-RFS (*p* = 0.012). The hazard ratios for intravesical BCG therapy were 0.31 and 0.29 for recurrence and high-grade recurrence, respectively, establishing it as a potent protective factor against both outcomes (both *p* < 0.001). By contrast, intravesical BCG therapy was not a significant factor of disease progression (Table S1).

## 4. Discussion

Here, we retrospectively reviewed and analyzed the data of 477 intermediate- and high-risk patients with treatment-naïve NMIBC and found that in the total cohort, the BCG arm demonstrated superior RFS and HG-RFS compared with the gemcitabine arm. Furthermore, in the propensity score–matched high-risk cohort, BCG was superior to gemcitabine in RFS and HG-RFS.

The multivariate Cox proportional hazards model revealed that intravesical BCG was a significant protective predictor compared with gemcitabine. It demonstrated a more pronounced relative risk reduction in the high-risk population than in the total cohort. Despite the inherent retrospective bias, where the BCG group initially had a higher proportion of T1 stage and high-grade tumors, the superior outcomes of BCG underscore its robust efficacy in suppressing recurrence and progression.

Standard guidelines highlight the superiority of intravesical BCG over chemotherapeutic agents in reducing the risk of cancer recurrence and progression. However, these guidelines are based on research on the efficacy of BCG over agents other than gemcitabine, mainly MMC and epirubicin [7,8,9,22]. Studies comparing the efficacy of intravesical BCG and gemcitabine in treatment-naïve high-risk patients are limited. In a randomized prospective study by Porena et al. of 64 patients with high-risk NMIBC (pT1 and/or G3 and/or CIS), BCG demonstrated a significantly lower recurrence rate than gemcitabine at a mean follow-up of 44 months (28.1% vs. 53.1%, *p* = 0.037) without disease progression in either group [23]. Conversely, Ali et al. enrolled 100 patients with high-risk NMIBC randomized to BCG or gemcitabine administration and reported no statistically significant difference in the recurrence (*p* = 0.2), progression (*p* = 0.06), or overall disease-free rates (*p* = 0.128) at 24 months of follow-up, although gemcitabine demonstrated a significantly more favorable AE profile than BCG (*p* = 0.002) [24]. In the present study, BCG demonstrated markedly superior oncological outcomes in the high-risk subgroup, with a median RFS not reached vs. 37.0 months for gemcitabine with a hazard ratio of 0.31 (*p* < 0.001) reflecting a more pronounced treatment effect than that reported by either prior study. These results provide robust real-world evidence supporting the continued use of BCG as the gold standard treatment over gemcitabine for treatment-naïve high-risk NMIBC. Given that the median number of maintenance instillations was 6.0 [2.0–12.0] in the BCG group and 8.0 [4.0–10.0] in the gemcitabine group, this cohort provides invaluable real-world evidence to validate the efficacy of maintenance protocols as recommended by current clinical guidelines. In terms of safety, intravesical gemcitabine was superior to BCG, which is consistent with the results of several previous studies [25,26].

Second TURBT has been recognized as a factor associated with improved survival outcomes, including RFS and PFS, in patients with NMIBC [27,28]. However, in the current study, second TURB was significantly associated with recurrence and high-grade recurrence in univariate analysis. However, it did not retain independent significance in the multivariate model. This finding suggests that second TURB was performed preferentially in patients with high-risk tumor or substantial tumor burden rather than representing an independent causal risk factor for recurrence.

We also performed an exploratory subgroup analysis comparing oncological outcomes and AE profiles between patients with BCG who received more than 12 instillations (*n* = 151) and the others (*n* = 210). Those who received more BCG instillations showed significantly better RFS and HG-RFS with comparable AE profiles (Figure S2 and Table S2).

This study has some limitations that warrant consideration when interpreting the results. Owing to its retrospective design, significant differences were observed in baseline characteristics between the BCG and gemcitabine groups regarding T stage, tumor grade, rates of muscle inclusion, single immediate instillation, second TURB, risk stratification, and follow-up period. To minimize the impact of these disparities, we constructed a propensity score–matched cohort of 273 patients from the 410 high-risk patients and conducted analyses on survival outcomes and risk factors. However, we could not perform PSM for the entire cohort of 477 intermediate- and high-risk patients due to the inherent channeling bias, which would have resulted in a non-representative sample of the real-world population. Therefore, the superior oncological outcomes of BCG in the entire cohort require careful interpretation, considering the baseline disparities. A substantial imbalance in the median follow-up duration between the BCG (45.0 months) and gemcitabine groups (24.0 months) also represents an important limitation of this study. Although time-to-event analyses account for censoring, this disparity may reduce the statistical opportunity to capture late progression events in the gemcitabine group, potentially underestimating the true long-term progression rate. Additionally, variations in clinical practice patterns between institutions were inherent in this multicenter study. These differences may have introduced unmeasured confounding factors not fully addressed in our analysis. Lastly, our study did not analyze second-line intravesical therapies administered after the failure of first-line treatment. Given that second-line therapy can significantly influence disease progression, this factor should be considered when interpreting the results related to disease progression.

## 5. Conclusions

This multicenter study of intermediate- and high-risk NMIBC populations demonstrated that intravesical BCG treatment offers significantly superior oncological benefits compared with gemcitabine in terms of RFS and HG-RFS after long-term follow-up. Intravesical BCG also resulted in significantly better survival outcomes in the propensity score-matched high-risk subgroup than in the total cohort. Further, it was a significant protective factor that reduced the risk of recurrence and high-grade recurrence in the total cohort and propensity score-matched high-risk subgroup. These robust outcomes, in intermediate- and high-risk NMIBC populations, were maintained despite a higher baseline proportion of T1 and high-grade tumors in the BCG cohort, further underscoring its clinical superiority. Intravesical BCG was associated with higher rates of irritative voiding symptoms and hematuria than gemcitabine. The findings of this study have direct clinical implications for guiding intravesical treatment decisions in patients with intermediate- and high-risk NMIBC, particularly in settings where BCG availability is limited. The real-world evidence presented here supports the prioritization of BCG over gemcitabine for high-risk patients. Furthermore, the comparative safety and efficacy data generated by this study may serve as a valuable reference for future research.

## Figures and Tables

**Table 1:** Baseline characteristics (*n* = 477).

	BCG (*n* = 361)	Gemcitabine (*n* = 116)	*p*-Value
Age (y), median [IQR]	70.5 [64.1–76.7]	70.7 [65.1–76.7]	0.721
Male sex (%)	300 (83.1%)	102 (87.9%)	0.243
Height (cm), median [IQR]	165.0 [160.8–170.0]	166.0 [161.8–171.0]	0.141
Weight (kg), median [IQR]	65.0 [58.0–73.0]	65.0 [59.5–72.8]	0.984
BMI (kg/m^2^), median [IQR]	24.2 [22.2–26.0]	23.8 [21.6–26.1]	0.374
Comorbidities (%)			
HTN	190 (52.8%)	62 (53.4%)	0.687
DM	110 (30.6%)	31 (26.7%)	0.161
COPD	12 (3.3%)	7 (6.0%)	0.272
CCVD	72 (20.0%)	21 (18.1%)	0.640
ESRD	5 (1.4%)	1 (0.9%)	1.000
Smoker (%)	141 (44.3%)	63 (54.3%)	0.082
T stage (%)			0.010
Ta	57 (15.8%)	33 (28.4%)	
Tis	15 (4.2%)	4 (3.4%)	
T1	289 (80.1%)	79 (68.1%)	
Tumor grade (%)			0.006
Low	30 (8.3%)	20 (17.2%)	
High	331 (91.7%)	96 (82.8%)	
Number of tumors, median [IQR]	3.0 [2.0–6.0]	4.0 [2.0–5.0]	0.729
Size of tumor (cm), median [IQR]	2.0 [1.2–3.0]	2.0 [1.0–2.8]	0.168
Rate of CIS positivity (%)	126 (35.0%)	35 (31.0%)	0.495
Rate of muscle inclusion (%)	275 (78.6%)	98 (87.5%)	0.039
Rate of single immediate instillation (%)	67 (18.6%)	35 (30.2%)	0.013
Rate of the second TURB (%)	212 (58.7%)	53 (46.1%)	0.023
AUA risk stratification (%)			0.007
Intermediate	42 (11.6)	25 (21.6)	
High	319 (88.4)	91 (78.4)	
Follow-up (months), median [IQR]	45.0 [26.0–60.0]	24.0 [15.8–32.2]	<0.001

BCG, Bacillus Calmette–Guérin; IQR, interquartile range; BMI, body mass index; HTN, hypertension; DM, diabetes mellitus; COPD, chronic obstructive pulmonary disease; CCVD, cardio-cerebrovascular disease; ESRD, end-stage renal disease; T stage, tumor stage; CIS, carcinoma in situ; TURB, transurethral resection of bladder tumor; AUA, American Urological Association.

**Table 2:** Intravesical therapy-related characteristics (*n* = 477).

	BCG (*n* = 361)	Gemcitabine (*n* = 116)	*p*-Value
Number of intravesical BCG treatments, median [IQR]	12.0 [8.0–18.0]	-	
Induction	6.0 [6.0–6.0]	-	
Maintenance	6.0 [2.0–12.0]	-	
Adequate BCG treatments (%)	286 (79.2%)	−	
Number of intravesical gemcitabine treatments, median [IQR]	-	14.0 [10.0–16.0]	
Induction	-	6.0 [6.0–6.0]	
Maintenance	-	8.0 [4.0–10.0]	
Duration of intravesical therapy (months), median [IQR]	12.9 [5.0–27.3]	10.4 [5.2–11.3]	<0.001
AEs of any grade (%)			
Dysuria/frequency/urgency	103 (28.5%)	17 (14.7%)	0.003
Hematuria	37 (10.2%)	3 (2.6%)	0.007
Abdominal pain/discomfort	8 (2.2%)	4 (3.4%)	0.497
Fever/urosepsis	3 (0.8%)	0 (0.0%)	1.000
Nausea/vomiting	0 (0.0%)	4 (3.4%)	0.003
Dizziness/fatigue/myalgia	16 (4.4%)	7 (6.0%)	0.462
AEs ≥ grade 3 (%)			
Dysuria/frequency/urgency	6 (1.7%)	0 (0.0%)	0.344
Hematuria	1 (0.3%)	0 (0.0%)	1.000
Abdominal pain/discomfort	0 (0.0%)	0 (0.0%)	1.000
Fever/urosepsis	0 (0.0%)	0 (0.0%)	1.000
Nausea/vomiting	0 (0.0%)	0 (0.0%)	1.000
Dizziness/fatigue/myalgia	1 (0.3%)	1 (0.9%)	0.428

BCG, Bacillus Calmette–Guérin; IQR, interquartile range; AE, adverse event.

**Table 3:** Propensity score matching balance in high-risk patients (BCG:Gemcitabine = 2:1).

	Before PSM (BCG, *n* = 319; Gemcitabine, *n* = 91)				After PSM (BCG, *n* = 182; Gemcitabine, *n* = 91)			
	BCG	Gemcitabine	SMD	*p*-Value	BCG	Gemcitabine	SMD	*p*-Value
Age (y), mean ± SD	69.9 ± 9.2	70.0 ± 9.2	0.004	0.889	69.9 ± 9.3	70.0 ± 9.2	0.005	0.887
T stage (%)			0.141	0.465			0.119	0.642
Ta	10.7	15.4			11.5	15.4		
Tis	4.7	4.4			3.8	4.4		
T1	84.6	80.2			84.6	80.2		
Tumor grade (%)			0.073	0.618			0.040	1.000
Low	1.3	2.2			1.6	2.5		
High	98.7	97.8			98.4	97.8		
Single immediate instillation (%)	17.2	30.8	0.321	0.007	29.1	30.8	0.036	0.888
Smoking history (%)	37.9	54.9	0.230	0.076	50.0	54.9	0.099	0.521

PSM, propensity score matching; SMD, standardized mean difference; SD, standard deviation; T stage, tumor stage.

**Table 4:** Risk factor analysis of RFS and HG-RFS in intermediate- and high-risk patients (*n* = 477).

	RFS				HG-RFS			
	Univariate		Multivariate		Univariate		Multivariate	
	HR (95% CI)	*p*-Value	HR (95% CI)	*p*-Value	HR (95% CI)	*p*-Value	HR (95% CI)	*p*-Value
Age	1.01 (0.99–1.03)	0.290	1.02 (1.00–1.05)	0.049	1.01 (0.99–1.03)	0.298	1.03 (1.00–1.06)	0.048
Male sex	1.40 (0.83–2.36)	0.209			1.18 (0.68–2.03)	0.559		
Smoking history	1.25 (0.89–1.76)	0.203	1.71 (1.12–2.60)	0.013	1.24 (0.85–1.81)	0.271	1.71 (1.06–2.77)	0.028
Single immediate instillation	1.05 (0.70–1.57)	0.828			1.15 (0.74–1.78)	0.546		
T stage								
Ta	Reference	-			Reference	-		
Tis	0.81 (0.31–2.09)	0.658			1.57 (0.57–4.33)	0.381		
T1	0.84 (0.55–1.23)	0.402			1.44 (0.83–2.50)	0.190		
High-grade tumor	0.93 (0.55–1.57)	0.784			1.58 (0.77–3.24)	0.215		
Number of tumors	1.10 (1.04–1.16)	0.001	1.11 (1.04–1.18)	0.001	1.12 (1.05–1.19)	0.001	1.12 (1.05–1.20)	0.001
Size of tumor	1.20 (1.06–1.35)	0.004	1.21 (1.06–1.37)	0.005	1.26 (1.10–1.44)	0.001	1.24 (1.07–1.43)	0.004
CIS	1.09 (0.76–1.55)	0.648			1.19 (0.81–1.76)	0.374		
Second TURB	1.485 (1.04–2.12)	0.029			2.09 (1.37–3.19)	<0.001		
Intravesical therapy								
Gemcitabine	Reference	-	Reference	-	Reference	-	Reference	-
BCG	0.40 (0.28–0.58)	<0.001	0.35 (0.23–0.54)	<0.001	0.43 (0.29–0.66)	<0.001	0.34 (0.21–0.55)	<0.001

RFS, recurrence-free survival; HG-RFS, high-grade recurrence-free survival; HR, hazard ratio; CI, confidence interval; T stage, tumor stage; CIS, carcinoma in situ; TURB, transurethral resection of the bladder tumor; BCG, Bacillus Calmette–Guérin.

**Table 5:** Risk factor analysis of RFS and HG-RFS in high-risk patients (*n* = 273; propensity matched cohort).

	RFS				HG-RFS			
	Univariate		Multivariate		Univariate		Multivariate	
	HR (95% CI)	*p*-Value	HR (95% CI)	*p*-Value	HR (95% CI)	*p*-Value	HR (95% CI)	*p*-Value
Age	1.01 (0.99–1.03)	0.476			1.01 (0.98–1.03)	0.554		
Male sex	1.49 (0.75–2.98)	0.258			1.32 (0.66–2.64)	0.437		
Smoking history	1.02 (0.67–1.57)	0.914			1.07 (0.69–1.68)	0.757		
Single immediate instillation	0.90 (0.56–1.43)	0.652			0.87 (0.53–1.43)	0.580		
T stage								
Ta	Reference	-			Reference	-		
Tis	1.53 (0.47–4.98)	0.477			2.03 (0.59–6.92)	0.260		
T1	1.33 (0.66–2.66)	0.424			1.58 (0.73–3.45)	0.249		
High-grade tumor	0.68 (0.17–2.75)	0.584			1.43 (0.20–10.29)	0.723		
Number of tumors	1.11 (1.03–1.19)	0.007	1.11 (1.03–1.19)	0.007	1.12 (1.04–1.21)	0.003	1.12 (1.04–1.21)	0.004
Size of tumor	1.17 (1.00–1.38)	0.055			1.25 (1.06–1.48)	0.008	1.25 (1.05–1.48)	0.012
CIS	1.09 (0.71–1.67)	0.707			1.05 (0.66–1.65)	0.842		
Second TURB	2.26 (1.35–3.76)	0.002			2.74 (1.56–4.83)	<0.001		
Intravesical therapy								
Gemcitabine	Reference	-	Reference	-	Reference	-	Reference	-
BCG	0.44 (0.28–0.68)	<0.001	0.31 (0.19–0.51)	<0.001	0.45 (0.28–0.72)	<0.001	0.29 (0.17–0.49)	<0.001

RFS, recurrence-free survival; HG-RFS, high-grade recurrence-free survival; HR, hazard ratio; CI, confidence interval; T stage, tumor stage; CIS, carcinoma in situ; TURB, transurethral resection of the bladder tumor; BCG, Bacillus Calmette–Guérin.

## Data Availability

The datasets generated and/or analyzed during the current study are not publicly available due to institutional privacy policies and patient confidentiality but are available from the corresponding author upon reasonable request.

## Supplementary Materials

The following supporting information can be downloaded at: https://www.mdpi.com/article/10.3390/jcm15103890/s1. Figure S1: Comparison of recurrence-, high-grade recurrence-, and progression-free survival rates between intravesical BCG and gemcitabine therapies in intermediate-risk patients (*n* = 67); Figure S2: Comparison of recurrence-, high-grade recurrence-, and progression-free survival rates between patients receiving > 12 vs. ≤12 intravesical BCG instillations (*n* = 361); Table S1: Risk factor analysis of PFS in total cohort (*n* = 477) and propensity score-matched high-risk cohort (*n* = 273); Table S2: Intravesical BCG therapy-related characteristics (*n* = 361).

## Abbreviations

The following abbreviations are used in this manuscript:

NMIBC	Non-muscle-invasive bladder cancer
TURB	Transurethral resection of bladder tumor
BCG	Bacillus Calmette–Guérin
AUA	American Urological Association
UC	Urothelial carcinoma
NS	Normal saline
CIS	Carcinoma in situ
AEs	Adverse events
RFS	Recurrence-free survival
PFS	Progression-free survival
IQR	Interquartile range
HG-RFS	High-grade recurrence-free survival
T stage	Tumor stage
CI	Confidence interval
