# Supplementary material for: Comparison of Long-Term Oncological Outcomes of Intravesical Bacillus Calmette–Guérin Versus Gemcitabine in Treatment-Naïve Non-Muscle-Invasive Bladder Cancer with Intermediate and High Risk: A Multicenter Retrospective Analysis
Source: J Clin Med. 2026 May 18;15(10):3890. doi: 10.3390/jcm15103890 (PMC13207305; doi:10.3390/jcm15103890)
Supplement: Supplementary file 1 [file jcm-15-03890-s001.zip › Table S2.pdf]

Supplementary Table S2. Intravesical BCG therapy-related characteristics (n = 361)

	**BCG instillation > 12 (n = 151)**	**BCG instillation ≤ 12 (n = 210)**	***p*-value**
Number of intravesical BCG treatments, median [IQR]	20.0 [15.0–24.0]	9.0 [6.0–11.0]	<0.001
Induction	6.0 [6.0–6.0]	6.0 [6.0–6.0]	0.324
Maintenance	14.0 [9.0–18.0]	3.0 [0.0–5.0]	<0.001
Adequate BCG treatments (%)	147 (97.4%)	139 (66.2%)	<0.001
Duration of intravesical therapy (months), median [IQR]	26.6 [18.0–35.8]	6.0 [2.5–11.4]	<0.001
AEs of any grade (%)			
Dysuria/frequency/urgency	77 (51.0%)	26 (12.4%)	<0.001
Hematuria	27 (17.9%)	10 (4.8%)	<0.001
Abdominal pain/discomfort	6 (4.0%)	2 (1.0%)	0.072
Fever/urosepsis	2 (1.3%)	1 (0.5%)	0.574
Nausea/vomiting	0 (0.0%)	0 (0.0%)	1.000
Dizziness/fatigue/myalgia	10 (6.6%)	6 (2.9%)	0.146
AEs ≥ grade 3 (%)			
Dysuria/frequency/urgency	2 (1.3%)	4 (1.9%)	0.477
Hematuria	0 (0.0%)	1 (0.5%)	1.000
Abdominal pain/discomfort	0 (0.0%)	0 (0.0%)	1.000
Fever/urosepsis	0 (0.0%)	0 (0.0%)	1.000
Nausea/vomiting	0 (0.0%)	0 (0.0%)	1.000
Dizziness/fatigue/myalgia	0 (0.0%)	1 (0.5%)	1.000

BCG, Bacillus Calmette-Guérin; IQR, interquartile range; AE, adverse event.
