# Supplementary material for: Comparison of Long-Term Oncological Outcomes of Intravesical Bacillus Calmette–Guérin Versus Gemcitabine in Treatment-Naïve Non-Muscle-Invasive Bladder Cancer with Intermediate and High Risk: A Multicenter Retrospective Analysis
Source: J Clin Med. 2026 May 18;15(10):3890. doi: 10.3390/jcm15103890 (PMC13207305; doi:10.3390/jcm15103890)
Supplement: Supplementary file 1 [file jcm-15-03890-s001.zip › Figure S1.pdf]

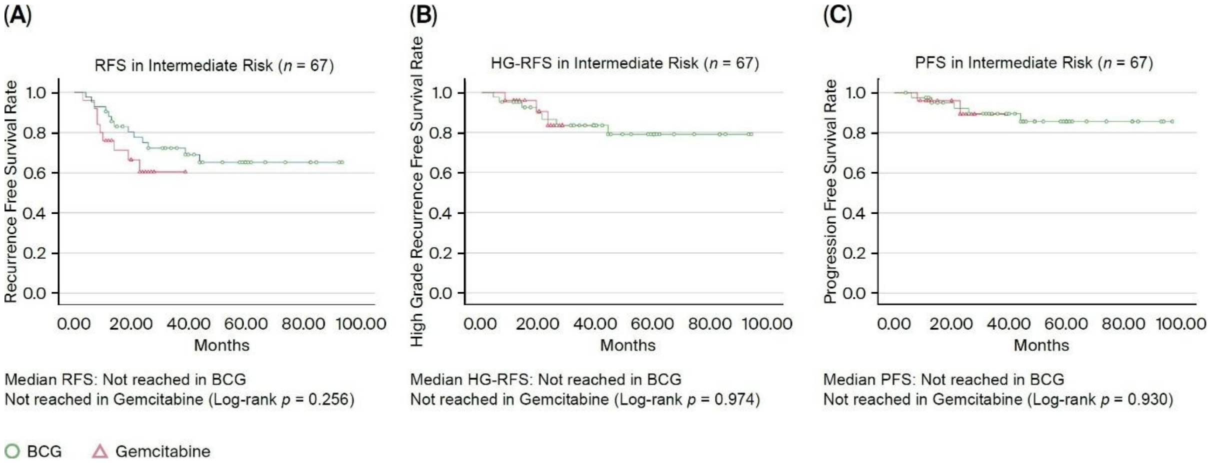

Supplementary Figure S1. Comparison of recurrence-, high-grade recurrence-, and progression-free survival rates between intravesical BCG and gemcitabine therapies in intermediate-risk patients (n = 67). Recurrence-free survival rate (**A**), high-grade recurrence-free survival rate (**B**), progression-free survival rate (**C**). RFS, recurrence-free survival; HG-RFS, high-grade recurrence-free survival; PFS, progression-free survival; BCG, Bacillus Calmette-Guérin.
