# Supplementary material for: Comparison of Long-Term Oncological Outcomes of Intravesical Bacillus Calmette–Guérin Versus Gemcitabine in Treatment-Naïve Non-Muscle-Invasive Bladder Cancer with Intermediate and High Risk: A Multicenter Retrospective Analysis
Source: J Clin Med. 2026 May 18;15(10):3890. doi: 10.3390/jcm15103890 (PMC13207305; doi:10.3390/jcm15103890)
Supplement: Supplementary file 1 [file jcm-15-03890-s001.zip › Table S1.pdf]

Supplementary Table S1. Risk factor analysis of PFS in total cohort (n = 477) and propensity score-matched high-risk cohort (n = 273).

	**PFS in total cohort (n = 477)**				**PFS in propensity score-matched high-risk cohort (n = 273)**			
	**Univariate**		**Multivariate**		**Univariate**		**Multivariate**	
	**HR (95% CI)**	***p*-Value**	**HR (95% CI)**	***p*-Value**	**HR (95% CI)**	***p*-Value**	**HR (95% CI)**	***p*-Value**
Age	1.02 (0.98–1.06)	0.289			1.03 (0.98–1.08)	0.248		
Male sex	6.02 (0.82–44.04)	0.077			3.49 (0.47–26.05)	0.222		
Smoking history	1.71 (0.86–3.42)	0.127	3.09 (1.36-7.04)	0.007	1.09 (0.46–2.57)	0.844		
Single immediate instillation	0.98 (0.43–2.27)	0.982			0.53 (0.18–1.56)	0.248		
T stage								
Ta	Reference	-			Reference	-		
Tis	0.80 (0.10–6.65)	0.837			1.20 (0.13–11.54)	0.875		
T1	1.03 (0.43–2.52)	0.941			0.86 (0.25–2.96)	0.813		
High-grade tumor	0.53 (0.22–1.28)	0.159			20.66 (0.00–129580329.0)	0.705		
Number of tumors	1.30 (1.16–1.45)	<0.001	1.34 (1.19-1.51)	<0.001	1.41 (1.19–1.67)	<0.001	1.48 (1.20-1.81)	<0.001
Size of tumor	1.45 (1.15–1.82)	0.002	1.29 (1.00-1.66)	0.047	1.33 (0.97–1.83)	0.079		
CIS	0.42 (0.17–1.02)	0.056	0.30 (0.12-0.76)	0.012	0.38 (0.13–1.12)	0.079		
Second TURB	2.84 (1.23–6.55)	0.014			3.67 (1.08-12.47)	0.037		
Intravesical therapy								
Gemcitabine	Reference	-	Reference	-	Reference	-	Reference	-
BCG	0.48 (0.22–1.03)	0.060	1.01 (0.38-2.67)	0.987	0.56 (0.22–1.41)	0.219	0.90 (0.25-3.32)	0.878

PFS, progression-free survival; HR, hazard ratio; CI, confidence interval; T stage, tumor stage; CIS, carcinoma in situ; TURB, transurethral resection of the bladder tumor; BCG, Bacillus Calmette-Guérin.
